# A revised definition for copal and its significance for palaeontological and Anthropocene biodiversity-loss studies

**DOI:** 10.1038/s41598-020-76808-6

**Published:** 2020-11-16

**Authors:** Mónica M. Solórzano-Kraemer, Xavier Delclòs, Michael S. Engel, Enrique Peñalver

**Affiliations:** 1 Palaeontology and Historical Geology, Senckenberg Research Institute, 60325 Frankfurt am Main, Germany; 2 Departament de Dinàmica de la Terra i de l’Oceà and Institut de Recerca de la Biodiversitat (IRBio), Facultat de Ciències de la Terra, Universitat de Barcelona, 08028 Barcelona, Spain; 3 Division of Entomology, Natural History Museum, and Department of Ecology & Evolutionary Biology, University of Kansas, Lawrence, KS 66045 USA; 4 Division of Invertebrate Zoology, American Museum of Natural History, New York, NY 10024 USA; 5 Instituto Geológico y Minero de España (Museo Geominero), 46004 Valencia, Spain

**Keywords:** Ecology, Zoology, Ecology, Environmental sciences, Evolution, Palaeontology

## Abstract

The early fossilization steps of natural resins and associated terminology are a subject of constant debate. Copal and resin are archives of palaeontological and historical information, and their study is critical to the discovery of new and/or recently extinct species and to trace changes in forests during the Holocene. For such studies, a clear, suitable definition for copal is vital and is herein established. We propose an age range for copal (2.58 Ma—1760 AD), including Pleistocene and Holocene copals, and the novel term "Defaunation resin", defined as resin produced after the commencement of the Industrial Revolution. Defaunation resin is differentiated from Holocene copal as it was produced during a period of intense human transformative activities. Additionally, the “Latest Amber Bioinclusions Gap” (LABG) since the late Miocene to the end of the Pleistocene is hereby newly defined, and is characterized by its virtual absence of bioinclusions and the consequent lack of palaeontological information, which in part explains the historical differentiation between amber and copal. Crucial time intervals in the study of resin production, and of the biodiversity that could be contained, are now clarified, providing a framework for and focusing future research on bioinclusions preserved in copal and resin.

## Introduction

Amber and copal originate from plant resins (both gymnosperms and angiosperms), which are progressively hardened through polymerization of their non-volatile compounds. Amber is well defined as a resin that has existed for millions of years^1^ and that can preserve microorganisms, plants, invertebrates, and vertebrates, largely since the Cretaceous period to the Neogene. Even more ancient ambers, frequently preserved in small quantities or even as traces, going back to the Late Triassic are known to have ensnared minute arthropods^2^. Traces of Palaeozoic amber are not known to contain inclusions. Due to their exceptional preservation potential, polymerized natural resins have been studied for centuries. Amber research provides data on the evolutionary history of organisms and their behaviour, that are otherwise rarely preserved in the fossil record, and on the ecology of forest ecosystems millions of years old (e.g.^3^). Conversely, the study of bioinclusions in copal and resin is especially relevant for understanding modern biodiversity, particularly in relation to Recent biogeographic or climatic changes as well as documenting recently extinct species or those that remain to be discovered^4–8^. Polymerized natural resins, such as amber and copal, can also be used as indirect proxies of atmospheric composition and climatic changes through time^9,10^.

Comparatively, the term copal is controversial as it has been used in varied contexts with different circumscriptions in different research fields; for example palaeontology, biology, geology, geochemistry, or archaeology^11–15^. This is owing to the fact that the term copal has been used without considering any typology of the resins based on their different states of polymerization or on their established ages. Copal and amber can be differentiated by FTIR analysis by observing precise exocyclic methylene bands^16^. In such analysis the intensity of the absorbance at 1600–1800 cm^−1^ and 1300–1500 cm^−1^ is related and conditioned by the oxidation history of the samples, which allows us to discriminate modern (less oxidized) resin samples from those that are fossilized^17^. Another possibility is the use of FT-Raman analysis by observing intensity of bands at 1646 and 1450 cm^−1^ and determining their ratio. Recent resins produce a higher peak at 1640 than at 1440 cm^−1^ (I1640/I1440 > 1, where I is the peak intensity), a ratio between 0.7 and 1.0 is established for copals and a ratio of 0.5–0.6 for ambers^18,19^. However, for both ratios no absolute or relative ages have been established with respect to these structural changes. Copal is considered an intermediate step in the formation between resin and amber^20^, as tree resin that is not completely fossilized or polymerized; this means that it has still not lost the main part of its volatile compounds. A further complication of language results from the fact that the word copal itself is derived from *copalli* in the language *Náhuatl*, used by the Aztec culture in Mexico, and explicitly means “resin”, in the sense of Recent resin or incense. The term is also misleading in that copal refers to both gymnosperm and angiosperm resins produced by different trees such as *Pinus* Linnaeus, 1753 (Gymnospermae: Pinaceae); *Agathis* Salisb., 1807 (Gymnospermae: Araucariaceae); *Protium* Burman, 1768 and *Bursera* von Jacquin ex Linnaeus, 1762 (Angiospermae: Burseraceae); *Liquidambar* Linnaeus, 1753 (Angiospermae: Altingiaceae); and *Hymenaea* Linnaeus, 1753 and *Copaifera* Linnaeus, 1762 (Angiospermae: Fabaceae)^21^. From these extant taxa, only *Hymenaea*, *Copaifera*, and *Agathis* have been able to produce copal (all three) and amber deposits (*Hymenaea* and *Agathis*). *Hymenaea* has been documented as the resin source for Mexican, Dominican, Venezuelan, and Ethiopian Miocene ambers^11,22–25^, and *Agathis* served as a source for New Zealand and Australian ambers^26,27^ as well as some Cretaceous ambers^28,29^. Today, the term copal is broadly used for all non-fossilized resins worldwide and lacking any true clarity in definition^8^, thus a differentiation is necessary between resins produced during the Pleistocene, Holocene, or in the yet-informal Anthropocene.

**Figure 1:**
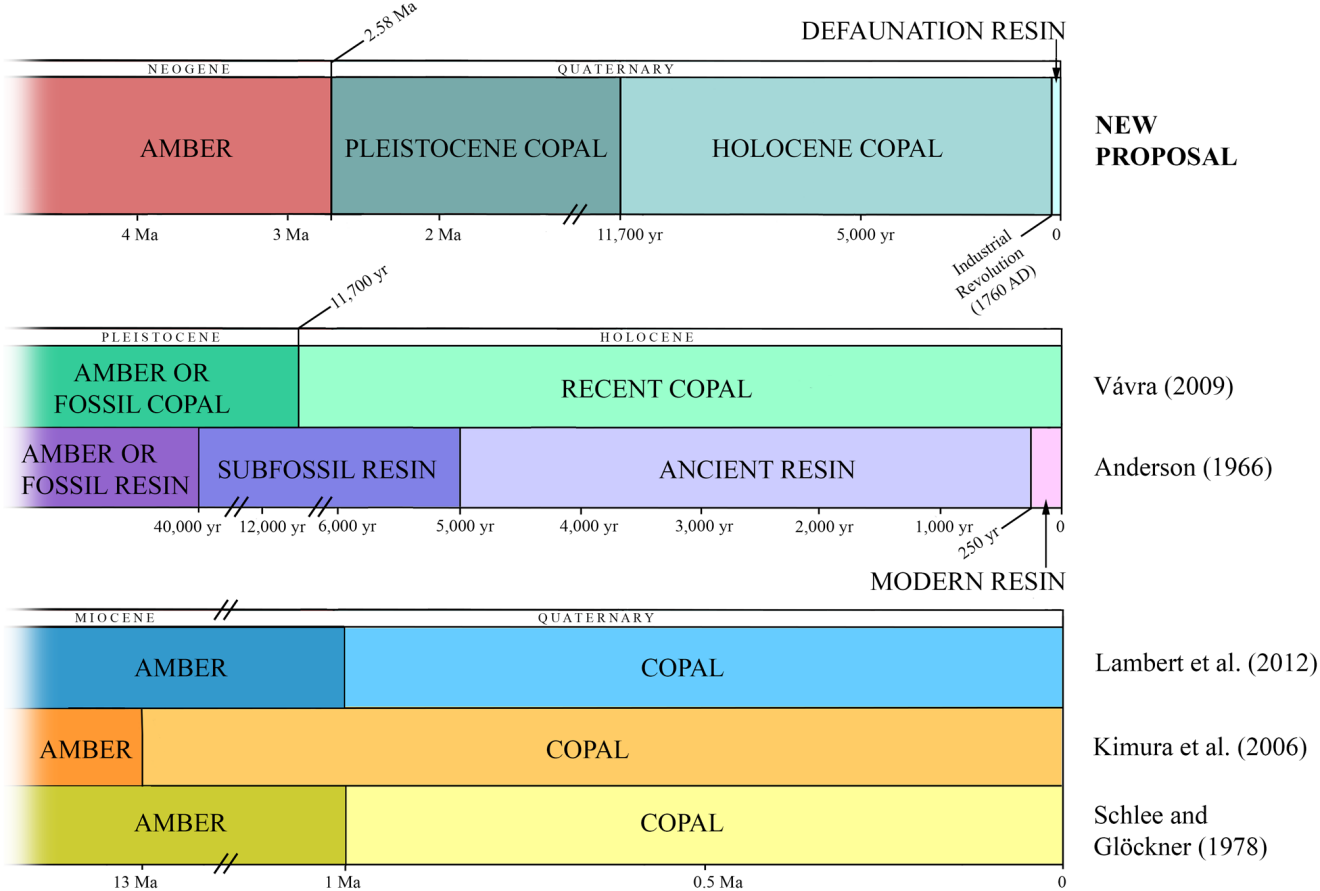
New proposal and different definitions of resin, copal, and amber from diverse authors for comparison. The time scales vary for the three graphics. Produced using Adobe Photoshop (www.adobe.com).

Chronologically, the term copal has been defined differently (Fig. 1). First, it was considered that resin younger than a million years old could be called copal^30^. Later, it was proposed as part of a scale based on absolute ^14^C dating^20^. In 1996, Anderson used the term “modern resin” for those resins less than 250 years old (today since 1770 AD, because the limit moves with time), “ancient resin” for resins between 250 and 5000 years old, and “subfossil resin” for resins with an age between 5000 and 40,000 years. He did not consider using the term copal for any of the polymerization steps of the resin. The term copal has been considered controversial^31^ but nonetheless distinguished between what was coined as “hard copals” or “copal” to refer to the resin found in the fruit pods of living *H*. *verrucosa* (p. 397)^31^. The polymerization process, which transforms copal into amber, has been considered to proceed exceedingly slowly, taking about 13 million years to become amber^32^; this concept implies that copal is younger than 13 million years and under this vague definition ambers from Peru and Borneo would have to be reclassified as copal. Copal has been also considered as immature resin that had failed to complete the fossilization process and classified as follows: those older than the Holocene were amber or fossil copal (> 11,700 years old) versus those younger than the Holocene were Recent copal^33^. Another work considered that resins of thousands of years to a million years in age better retain their original molecular structure and are therefore better termed as copal^34^; however, there is no evidence for this, and the fossilization process of resin remains poorly understood. Recently, Colombian copal dated with ^14^C analysis from post-World War II to approximately 10,600 years old was considered (sub) Recent (Anthropocene) copal^7^.

Disregarding processes involving reworking, the age of a resin-bearing sedimentary deposit is one criterion for separating resin from copal, including also its occurrence as material buried at deep levels within soils. However, the stratigraphic history of most copal deposits, with the exception of Madagascar^8^, remains to be studied and the ages of different samples are mentioned in the literature under quite broad ranges (see the Supplementary Table S1). Malagasy copal (sometimes called “Madagascar copal” in the literature) originated from trees of *Hymenaea* and has often been limited to between 10,000 years old and 5 million years old (e.g.^35,36^); however, it is now known that this resin, in the Malagasy soils, can only be preserved for an estimated maximum of 300 years before complete deterioration^8^. The use of ^14^C dating for copal with bioinclusions is not common. Samples of Kenyan and Tanzanian copals dated by ^14^C originated from resin exuded around 1960 AD^37^. However, in Eshnunna (Iran) and in Dendera (Egypt) similar East African copal with bioinclusions were found in archaeological sites of ages around 2500–2000 BC^38,39^. Dominican copal can reach an age of > 36,000 years BP (Conventional radiocarbon age, hereafter BP)^40^ and two pieces of Dominican copal dated as 10,820 years BP and 1700 years BP, respectively, have also been mentioned in the literature^41^. The copal from Japan, known as Mizunami copal, and which contains bioinclusions, can reach an age of 33,100 years BP^42^. New Zealand copal has been analysed by ^14^C reaching an age of 2000–37,000 years BP, however these do not contain bioinclusions^43^. One piece of Colombian copal has been dated as 10,600 years BP^44^, and one analysed piece of Sulawesi copal (Celebes, Indonesia) obtained an age of 4000 years BP^45^. One important problem is the fact that for different copals from diverse regions no occurrences of stratigraphically well-dated (i.e., with index fossils) copals exist^30^. Thus, most of the copal used for scientific studies has been arbitrarily allocated from the Pleistocene to the Present (spanning at least 2.58 million years).

The youngest known amber deposits are the Amazonian amber in Peru^46^ and the Bornean amber in the Indo-Australian Archipelago^47^, both of which are considered to be 12 million years old. Both have only a few recorded bioinclusions, from which only one species has been formally described in Bornean amber^47,48^, and a further two in Amazonian amber^49,50^. The resin-producing tree of the Amazonian amber is still unknown but it was an angiosperm^46^. *Shorea* sp. (Angiospermae: Dipterocarpaceae) has been proposed as a possible tree source for Bornean amber; this amber can be found in different geological contexts ranging from the middle Miocene to Pleistocene, although the “young amber” pieces are most probably reworked from older beds^47^.

Countries and regions where copal has been reported are: Brazil, Colombia, Dominican Republic, East Africa (Kenya, Somalia, Tanzania, Zanzibar), West Africa (Angola, Benin, Congo, Gabon, Ghana, Guinea, Nigeria, Sierra Leone), Madagascar, Mauritius, Seychelles, Borneo, Indonesia, Japan, Malaysia, Philippines, Sumatra, and New Zealand^1,31,51^. Descriptions of the biota included in copal have been mainly focused on copal from Colombia, East Africa (mainly Tanzania, Zanzibar), and Madagascar. From East African and Malagasy copals about 120 species have been described in more than 80 publications^8^. More than 20 publications deal with the description of new species in Colombian copal (e.g.^44,52^), some of which have since been synonymized with living species^53^, while at least one taxon has been described in copal from the Dominican Republic^41^. It is noteworthy that from the tens of tons of copal extracted from the Pleistocene and Holocene deposits of New Zealand, no publication on its palaeobiological content has been undertaken.

We deal here with two problems: (1) bioinclusions in copal are described and published without accurate dating (absolute or relative), and (2) the term copal is not clearly defined, however extensively used. Accurate dating applying radiocarbon analysis is currently widely affordable, allowing us to address the first challenge, and from those results a suitable and useful definition of the term copal is proposed herein.

## Results

### Pleistocene copal, Holocene copal, and Defaunation resin

We propose here a new definition of the term copal: *ancient resin having an age between 2.58 Ma and 1760 AD.* In addition, we propose the use of the terms: *Pleistocene copal* (2.58–0.0117 Ma), *Holocene copal* (0.0117 Ma—1760 AD), and as a novel term: *Defaunation resin*, defined as *resin exuded after 1760 AD*, which is the starting point of the Industrial Revolution^54^ (Fig. 1) and coincides with the Great Acceleration presented by Steffen et al.^55^ from 1750 to 2010. The term is based on the defaunation concept according to Dirzo et al.^56^: “The term defaunation, used to denote the loss of both species and populations of wildlife, as well as local declines in abundance of individuals, needs to be considered in the same sense as deforestation, a term that is now readily recognized and influential in focusing scientific and general public attention on biodiversity issues.” (p. 401^56^).

For the use of this new terminology and for the study of evolution of exuded resin to amber, the information about when the resin was produced or buried in a geological deposit is needed. With such information it is possible to classify resin and fossil resin as follows (our proposal):

1. *Defaunation resin*; resin produced after 1760 AD, early buried or promptly collected before degradation in the soil surface, or even before it reached the soil. In these samples it is possible to study faunal changes or other abiotic changes (e.g., atmospheric changes), globally or in a single region, caused by human activities. It could contain extinct and living species, mainly the latter, but also species still not yet discovered living in the ecosystem (particularly true for insects as in many biodiversity hotspots much of the modern fauna remains to be documented scientifically). The cutoff of 1760 AD was chosen based on the hypothesis that a decline of biodiversity and other severe environmental changes are caused by human activities, commencing with the Industrial Revolution. Determination of age can be done with ^14^C analysis.
2. *Pleistocene copal* (2.58–0.0117 Ma) and *Holocene copal* (0.0117 Ma—1760 AD); resin produced during the Quaternary and older than 1760 AD; the limit between both corresponds to the end of the Younger Dryas stadial, from ~ 12.95 to 11.6 kyr cal BP, representing an abrupt Northern Hemisphere cooling episode and is considered the end of the last glacial period. They also could contain extinct and living species, mainly the former. Determination of the age can be done by ^14^C analysis for pieces not older than 50,000–60,000 years BP. For copal pieces, principally Pleistocene copal, the geological context should also be studied. In this form, we avoid the confusion caused by previous definitions (Fig. 1), principally when bioinclusions are taxonomically studied.
3. *Amber*; which is resin from the Pliocene or older (> 2.58 Ma). If bioinclusions are present, most likely they correspond to extinct species because of its antiquity, and they can offer a baseline to study the evolution of organisms. In this case, age can be determined by stratigraphical/palaeontological studies and by absolute dating (e.g.^57^).

For the present study, the oldest piece of copal with bioinclusions we tested was 2180 ± 30 years BP from Colombia, followed by a 1050 ± 30 years BP piece of copal from Tanzania. Copal without bioinclusions from Congo was dated as 5480 ± 35 years BP and from New Zealand as 41,370–30,000 years BP, according to our samples and Lambert et al.^43^ (see the Supplementary Table S1).

### Latest Amber Bioinclusions Gap (LABG)

**Figure 2:**
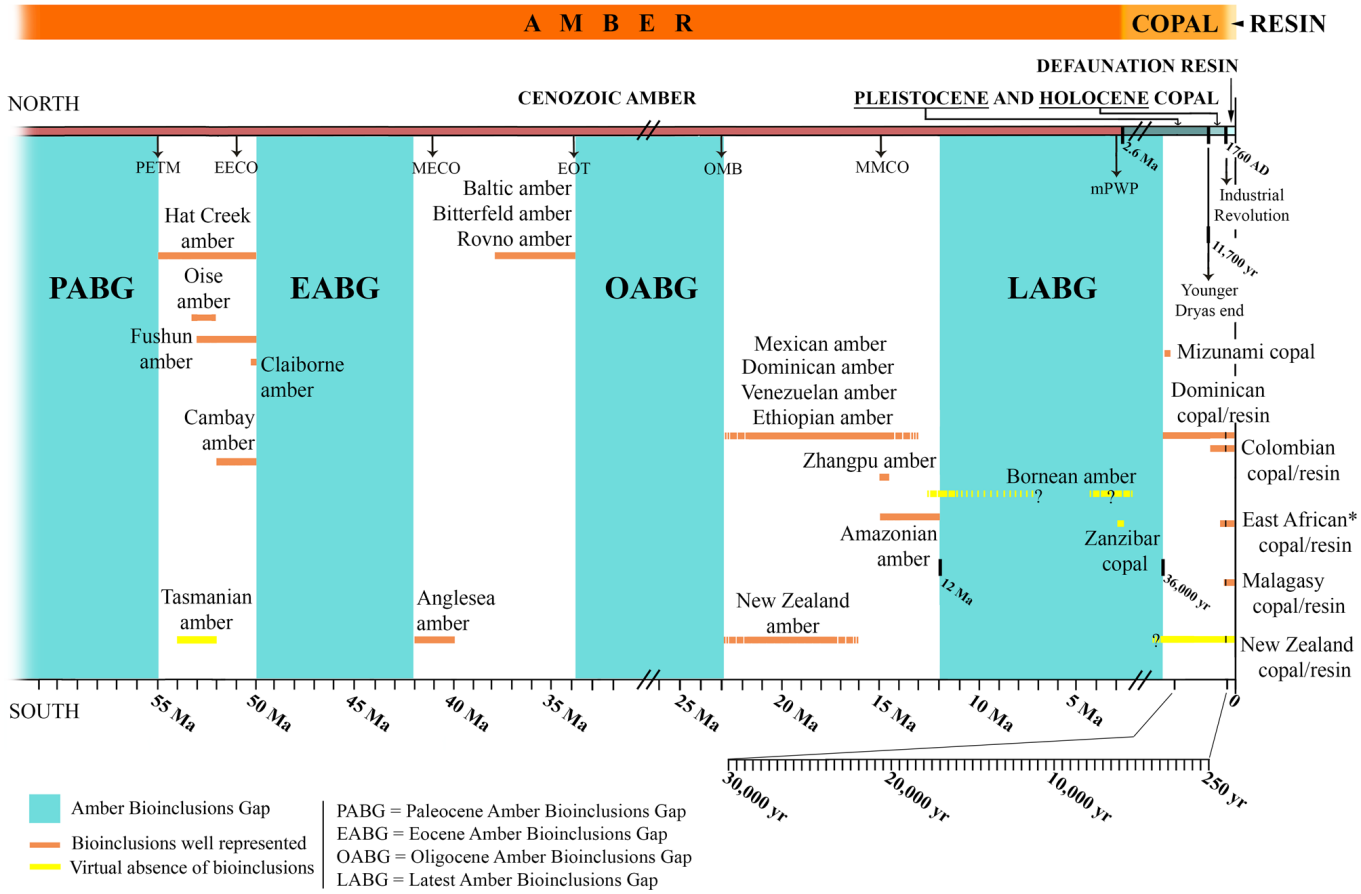
Representation of the Amber Bioinclusions Gaps (ABGs) during the Cenozoic, including the Latest Amber Bioinclusions Gap, the newly defined Defaunation resin, and recalibration for the terms copal and amber. This graphic only contains the main bioinclusions gaps that imply periods of several millions of years; their limits are approximate in relation to the broad age estimates of the main fossiliferous ambers. Some ambers with ages poorly known and under debate (e.g., Sicilian amber), and some others with a virtual absence of bioinclusions, have not been represented. Only some representative copals dated with ^14^C are included here (see the Supplementary Information for the references on which the amber ages are based). Supplementary abbreviations: EECO = Early Eocene Climatic Optimum, EOT = Eocene–Oligocene Transition, MECO = Middle Eocene Climatic Optimum, MMCO = Middle Miocene Climatic Optimum, mPWP = mid-Pliocene warm period, OMB = Oligocene–Miocene Boundary, PETM = Paleocene–Eocene Thermal Maximum. Produced using Adobe Photoshop (www.adobe.com).

We define here the “Amber Bioinclusions Gaps” (ABGs) during the Cenozoic and with special focus the “Latest Amber Bioinclusions Gap” (LABG, Fig. 2) for the purposes of the present research. We consider herein the concept of a virtual absence of *bioinclusions* during a time period of several millions of years instead of the virtual absence of ancient *resin-bearing deposits*. This distinction emphasizes, from a paleontological point of view, the relevance of the information that ancient resins provide, based on their bioinclusions, to ancient forest ecosystems.

For example, the Bornean amber and New Zealand copal deposits (Fig. 2) provide little knowledge in this respect. The LABG is flanked between the oldest copals with bioinclusions, a Pleistocene copal from the Dominican Republic around 36,000 years BP^40^, and the youngest amber with some bioinclusions, which is 12 million years old and comes from Peru^46^. The LABG is not only characterized by an almost complete absence of bioinclusions in both copal and amber but also by the scarcity of fossil resin deposits during this temporary interval worldwide. This absence is probably due to the natural response to a general tendency of reduction of the global temperature and *p*CO_2_ during the Pliocene–Pleistocene^58–60^, after the warmer mid-Miocene Climatic Optimum (MMCO, 14.5–17 Ma). The MMCO covered an average increase in global temperature of 3–4 °C coincident with oceans becoming more depleted in ^12^C relative to ^13^C than at any other time in the past 50 Ma, and resulting from volcanic degassing, global warming, and sea-level rise^61^. This correlation is also observable during the middle Eocene Climatic Optimum (MECO) and the early Eocene Climatic Optimum (EECO), with high global temperatures, high CO_2_ levels, and an increase in precipitation probably resulting from elevated volcanic emissions^62–64^.

As its name indicates, the LABG (~ 36,000 years BP—12 Ma) is the most recent gap in the history of resin production and includes the geological time interval from the late Miocene to Pleistocene. It is here defined within a strict time interval; however, this must be considered to have a certain degree of flexibility because new older copals or younger ambers with bioinclusions may be found or the age of known deposits may be re-studied and re-dated. In any case, this gap can cover, in part, the origin of the historical differentiation between amber and copal, provides a convenient circumstance to define these terms, and justifies the amber-cut-off in the Pleistocene. Three other main Cenozoic ABGs, extending several millions years each, can be establish during the Palaeocene (PABG), Eocene (EABG), and Oligocene (OABG) (Fig. 2). The diverse potential causes of them, surely not only main episodes of change in the global temperature, constitute an important topic of study, but are beyond the scope of the present research.

## Discussion

Copal and amber are organic materials in which a continuous polymerization of organic hydrocarbon molecules changes the resin into amber^65^. According to Anderson and Crelling^66^, diagenetic alteration and the degree of polymerization/maturation are indicators of relative age; accordingly, the classification of resin, copal, and amber should be based on the ensemble of chemical composition. However, hitherto there has not been a technique using the loss of volatile compounds as an indicator of age for fossil or sub-fossil resins, this is probably because of the complexity of these compounds^14^ and the diversity of taphonomic processes, including the geological context (fossil diagenesis). The impact of thermal alteration on natural resins was established based on terpenoid condensation parameters, which can be used to define the type of amber deposits^67–70^. However, thermal analysis only allows for a relative differentiation between ambers and less ancient resins^71^, instead of an absolute age.

Copal and amber can also be differentiated by FTIR spectroscopy by observing precise exocyclic methylene bands^16^. Copal can be differentiated by weak bands at 3048 and 1642 cm^−1^, and by an intense band at 887 cm^−1^. In the case of ambers these bands are weak or absent^72^. Thus, the diagenetic alteration can easily be used to differentiate amber from young resin but not to distinguish clear definitions of copal and resin, or to delimit an absolute age for amber. Furthermore, some resins have the property of polymerizing quite quickly, e.g., *Hymenaea* spp. resin can harden within days due to the position of the carboxylic group in iso-ozic acid at the C4 position and the enantio configuration^73^ giving the impression of being older than it actually is^8,15^. Recently, Stach et al.^74^ attempted to correlate microhardness of copal and amber with age; unluckily, these authors used uncorroborated and wrongly cited ages for the copals tested, apart from the fact that the provenance of Malagasy and Tanzanian copals is frequently uncertain^8^. With this information, they argued an older age for Malagasy copal than that for the Colombian and Tanzanian copals.

Hitherto, only ^14^C can date the absolute age of Defaunation resin, and Holocene and Pleistocene copals not older than 50,000 years. The oldest date that can be reliably measured by ^14^C dating is around 50,000 years, although special preparation methods occasionally permit accurate analyses of older samples. In the literature, and in the results of the present work, the youngest resin piece dated was about 60 years BP. The oldest copal with bioinclusions reached about 36,000 years BP^40^, and a piece without bioinclusions was measured at about 41,000 years BP (see the Supplementary Table S1). Thus, our results highlight the possibility to use radiocarbon dating for the study of bioinclusions in Pleistocene and Holocene copals or in Defaunation resin.

**Figure 3:**
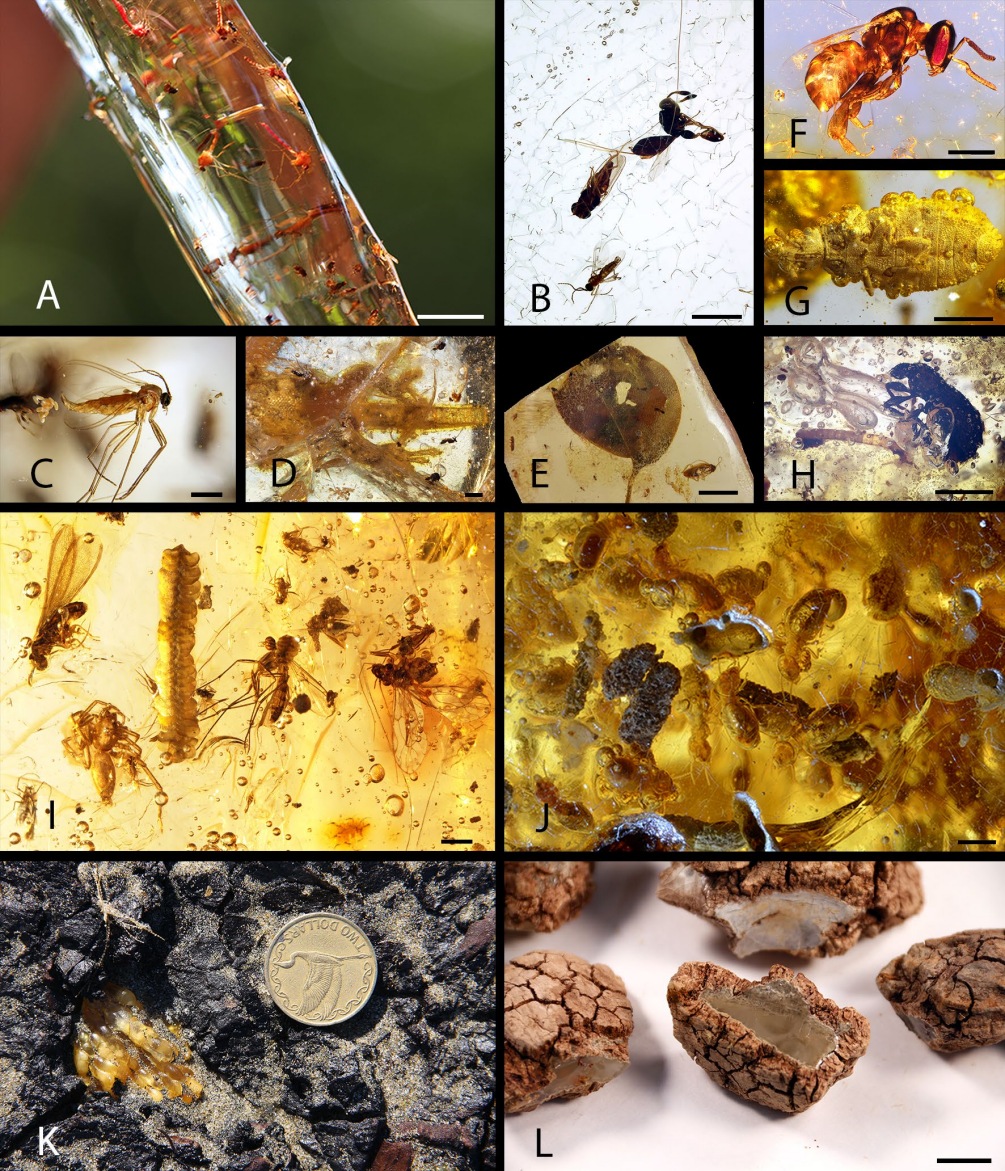
Bioinclusions in Defaunation resin and Holocene copal from different places. (**A–E**) Defaunation resin from trees of *Hymenaea verrucosa*, Madagascar. (**A**) swarm of non-biting midges (Diptera: Chironomidae). (**B**) piece containing diverse bioinclusions (Hymenoptera: Chalcidoidea, Diptera: Brachycera, and Diptera: Nematocera: Sciaridae). (**C**) fungus gnat (Diptera: Sciaridae) that laid eggs during its death. (**D**) lizard body portion. (**E**) *H*. *verrucosa* leaf and different insects as bioinclusions. (**F–G**) Holocene copal from *H*. *verrucosa*, Tanzania. (**F**) stingless bee (Hymenoptera: Apidae). (**G**) flat bug (Hemiptera: Aradidae). (**H**) Defaunation resin (ca. 200 years old; radiocarbon dating) from tree *Agathis australis*, New Zealand, with different bioinclusions (Coleoptera and larva indet.). (**I–J**) Holocene copal from trees of *Hymenaea courbaril*, Colombia. (**I**) diverse bioinclusions [Arachnida: Araneae, Insecta (Psocoptera, Isoptera, Diptera), and Plantae: Marchantiophyta]. (**J**) termite (Isoptera) nest. (**K**) in situ Pleistocene copal from tree *Agathis australis*, Baylys Beach, New Zealand (coin diameter 26.5 mm). (**L**) Defaunation resin most probably from trees of *A. lanceolata*, Parc de la Rivière Bleue, New Caledonia. Scale bars: (**A**) 5 mm, (**B**–**D** and **F**–**J**) 1 mm, (**E** and **L**) 1 cm.

**Figure 4:**
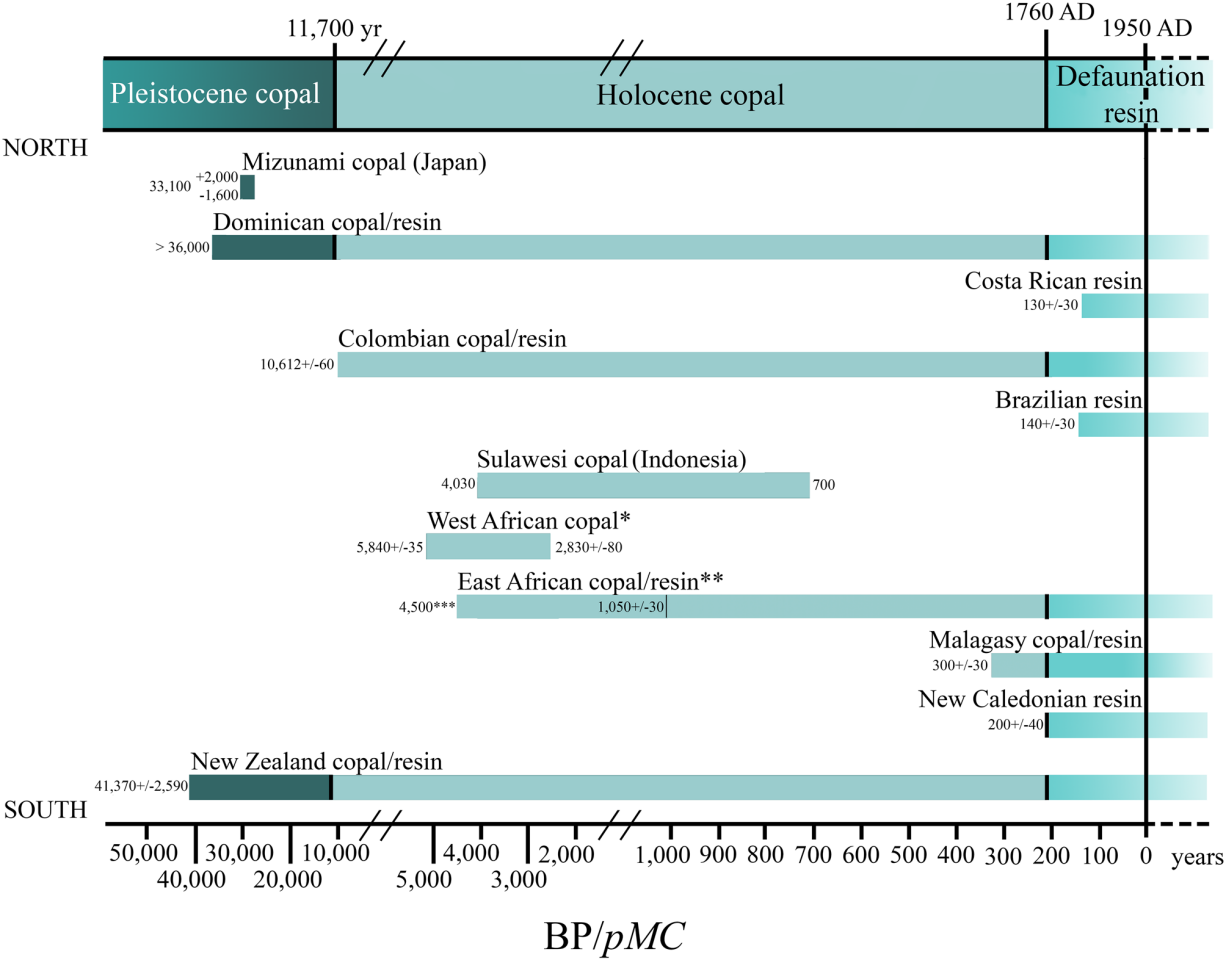
Minimum and maximum values given to the different copals tested by radiocarbon analysis (^14^C). Not the whole time span is covered by ^14^C results, see the Supplementary Table S1. *West African copal includes copals from Angola and Congo. **East African copal includes Tanzanian and Zanzibar copals. ***Dating of 4500 years come from archaeological data mentioned by Meyer et al*.*^38^ and not from ^14^C as all others. Produced using Adobe Photoshop (www.adobe.com).

Pleistocene and Holocene copals and Defaunation resin provide access to biotas that are otherwise challenging to capture in the fossil record (Fig. 3A–L). The fauna (mainly arthropods) and flora preserved in resin and copal allow for studies on the composition and change of particular biotas in threatened terrestrial ecosystems, particularly those today experiencing increased biodiversity losses. For example, the ants in Colombian copal were studied by DuBois and LaPolla^75^, unfortunately without first analysing the age of the studied material. However, as a principal result, these authors concluded that this fauna clearly represents a picture of the ant fauna prior to intense human transformation in South America. This kind of result is important to be re-evaluated, and including taphonomic studies^6^, in order to consider bias in the compared faunas. However, regardless of age, Pleistocene and Holocene copals and Defaunation resin provide access to biotas that lived at the beginning of the Industrial Revolution or under pre-industrial conditions (Fig. 4), allowing comparisons with those living arthropod communities altered by humans.

The term amber, and also when including a general indication of its age—e.g., Miocene amber, Eocene amber, etc.—is well defined and commonly used in varied research fields such as palaeontology, biology, geology, geochemistry, and archaeology. By contrast, the term copal has been used ambiguously, mainly because the age is not properly investigated and because the different concepts for copal and resin that have been established often do not take into account the geologic time scale, the deforestation and defaunation within the Anthropocene in which the sixth extinction is now occurring^56,76,77^, and the relevance of the age span for faunal studies.

Following Delclòs et al.^8^, copal samples should be dated using ^14^C, particularly since copal from a single country can vary in age (see the Supplementary Table S1). Accordingly, the term copal can be used in a manner like that is done for amber, i.e., with a specific reference to its age, as in the format “[epoch] copal” and then location (e.g., Holocene copal from Colombia). This is especially important for pieces of, for example, Colombian and Dominican copals which contain bioinclusions and that are in the age limit of the Holocene^7^, and for those pieces that were previously dated from the Pleistocene or the Holocene before 1760 AD^40,41,52^. Furthermore, this terminology will help to differentiate resin occurrences of different ages from the same geographical region (Defaunation resin vs Holocene copal, see the Supplementary Table S1, Fig. 4).

The Quaternary is divided into two epochs, Pleistocene and Holocene. The Pleistocene ranges from 2.58 Ma to 11,700 years ago and the Holocene spans from after the last major ice age^60^ to the Present. Within the Holocene, the Meghalayan stage has been recently ratified by the International Commission on Stratigraphy (ICS) as 4200 years BP to 1950 AD; however, this has quickly come under debate^78^. The recently proposed, but as-of-yet unratified, Anthropocene is that span of time when humans dominated and globally altered most natural processes. Proposals for the beginning of the Anthropocene range from the Megafauna extinction 50,000 years ago to as recent as 1964 AD or 1945 AD with the rise of nuclear weapon detonations and persistent industrial chemical products^79,80^. However, it remains an unofficial geological unit. Neither the ICS nor the International Union of Geological Sciences (IUGS) have officially approved the Anthropocene as a recognized subdivision of geologic time and how it should be demarcated remains a matter of discussion^81–84^. On this basis, Defaunation resin was produced within the Anthropocene in a different time relative to the remainder of the Holocene. Accordingly, resin produced during this timeframe must be distinguished, not in a geological context, but in the context of the dramatic alteration of Recent terrestrial ecosystems. One of the proposals for the beginning of the Anthropocene considers changes in the eighteenth century such as rates of vertebrate extinctions caused by human activities, such as the spread of agricultural development, deforestation, the Columbian Exchange of Old Word and New World species, and the Industrial Revolution^81^. In addition, the Great Acceleration was characterized with trends starting from 1750 AD, including tropical forest loss, domesticated land development, terrestrial biosphere degradation, and/or rapid increase of the human population^55^, all relevant variables for the study of arthropods in resin from tropical forests produced before and after the Industrial Revolution. The year 1610 AD has also been proposed as a starting point for the Anthropocene^79^, coinciding with the biotic change showed by Ceballos et al.^77^ and fits more closely with Crutzen and Stoermer’s original proposal to place the beginning of the Anthropocene at 1760 AD, which has been enduringly popular and useful^79^. Thus, 1760 is an appropriate starting point for the here defined “Defaunation resin”. Although our other terms are based on epochal names, given the on-going debate surrounding the Anthropocene and that it remains unratified, we believe the starting point and terminology suggested here is both useful and uncontroversial.

The Anthropocene can be differentiated clearly from the Holocene, with human activity as the causative agent^81,85–87^. In this regard, biologists are speaking about “Anthropocene defaunation” for the loss of species, populations, and abundance of individuals in the global biota^56^. Anthropocene defaunation extends to the insect fauna with rates of extinction mentioned in the literature as dramatic as 40% of the world’s species in the last few decades^88^. In this regard, the new term “Defaunation resin” from 1760 AD to the present is of great relevance, taking into account the recording function that the resin has in this context.

In their study, Sánchez-Bayo and Wyckhuys^88^ presented data for extinct species from studies carried out 50 years ago or earlier, while most of the pertinent copals and resins produced are older (see the Supplementary Table S1). Furthermore, bioinclusions in copals and resins represent an unbiased sample of organisms, with respect to human-disturbed habitats or human sampling, that lived in close relation with the resin-producing tree^6^, and consistently from understudied ecosystems such as lowland forests. In contrast, most entomological collections and sampling protocols are traditionally focused on one group of organisms, representing the taxonomic research interests of the curators and investigators involved. There are, however, notable exceptions where sampling is done more generally to gather a broader ecological perspective on a given area, but these are the minority across the vast entomological research collections of the world. With the aforementioned in mind, we propose here a new terminology. The use of these terms: (1) will clarify and allow studies of organisms included in resin, and copal, the importance they deserve as an archive of historical information, (2) will highlight the need for ^14^C analysis of each resin and copal piece studied having bioinclusions, (3) will contribute to the study of the arthropod fauna in countries with a top global conservation priority for their high levels of endemism and threat, such as Madagascar, East Africa, and Colombia^89–91^.

With the establishment of the LABG (12–0.0117 Ma), the distinction of copal (2.58 Ma—1750 AD) and amber (> 2.58 Ma) can be better understood from a historical point of view. Most likely, because there is a gap between the late Miocene and Pleistocene in resin production and/or resin preservation, principally resin with bioinclusions, the term copal came to apply through time for resin produced after Miocene amber. The LABG can show the age span in which copal and amber with bioinclusions can be found, and the time span from which such bioinclusions can reveal something about the biodiversity at the time the resin was exuded. Hitherto, only the amber from the Indo-Australian Archipelago has been presumed to date from the Miocene to the Pleistocene; however only a few bioinclusions have been found and these are mostly from the middle Miocene. One hypothesis is that probably the environmental conditions during the late Miocene and Pleistocene did not promote the production of resin in sufficiently large amounts to produce amber or copal deposits rich in bioinclusions. Resin production appears to have decreased considerably, at least not to promote the formation of this type of geological deposits in the late Miocene. This is related to the increase in aridity and in temperature gradients globally, which promoted important changes in terrestrial ecosystems^92^. These changes are the consequence of an atmospheric CO_2_ drop^93–95^. Furthermore, the Pliocene, Pleistocene, and some periods during the Holocene are characterized by persistent succession of glacial-interglacial cycles^58,59,96^. During these important periods of global ecological changes, tropical forests were dynamically shifting in position and the extent of their areas of coverage^97^, and probably did not form large amber or copal deposits as a consequence. According to Novick et al.^98^, resin production, as a defence strategy, can be correlated with beetle activity, which increases under warm climates corresponding to elevated *p*CO_2_. However, aside from insect attacks, disease, traumatic wounding from fires and storms, tree architecture, and local soil conditions are also significant factors for the production of resin emissions or for its preservation^8,99,100^. Thus, more research focused on this topic is needed to better understand the abiotic and biotic processes that condition the mass exudation of resin and its fossilization.

The age of Pliocene—early Pleistocene for the Dominican amber coming from the Yanigua Formation (El Valle region) proposed by Braga et al.^101^ does not match the here defined “LABG” because the amount of bioinclusions is comparable with the most important amber deposits. However, that age for this amber is highly debated^102^ owing to its importance for the palaeobiogeography of the Caribbean fauna and for molecular clock calibrations; additional geological studies must be done.

Amber Gaps and Amber Bioinclusions Gaps in the history of resin production from the Triassic to the Miocene are not rare^100^. Beside the establishment of the LABG, we herein name for the first time the Cenozoic Gaps as Oligocene Amber Bioinclusions Gap (OAGB), Eocene Amber Bioinclusions Gap (EABG), and Paleocene Amber Bioinclusions Gap (PABG) (Fig. 2). In the future, Mesozoic Amber Bioinclusions Gaps could be defined, e.g., Jurassic Amber Bioinclusions Gap since a few Jurassic amber-bearing outcrops are known but yielding amber pieces without plant and animal inclusions^103–105^.

The new definition of the term copal proposed herein and the new terms Pleistocene copal, Holocene copal, and Defaunation resin discussed and/or proposed herein clarify some time intervals in the study of resin production, and of the biodiversity that ancient and modern resin could contain in the form of bioinclusions (Fig. 2). The new definitions are of long-term use, since the Pleistocene and Holocene are well established as geological epochs. Defaunation resin falls within the informal Anthropocene Epoch (noting that its definition, if ratified, could change), and is established as starting at 1760 AD with the Industrial Revolution. Thus, the focus here is to establish a term that permits the study of organisms included in copal or in resin in a geographical and historical context that allows for a better understanding of biodiversity loss in tropical environments.

Because the term copal is widely used in combined names indicating the geographical origin of this substance, e.g., “Colombian copal” or “Malagasy copal”, it should continue to be used. However, in technical studies it must be accompanied with both its age and geographic provenience, e.g., “Holocene Colombian copal” and “Defaunation Colombian resin”. We standardize here definitions for future studies using modern and fossil taxa that have equal fossilization potential. Resin produced during the ongoing informal epoch of the Anthropocene — the “Defaunation resin” — is important and should be studied because it could contain extinct species due to the high rate of deforestation and its associated diversity loss during the last 300 years. We are losing biodiversity everywhere, principally in tropical environments such as the lowland forests in those countries where copious resin production occurs. Perhaps, if this intense deforestation continues, the biota included in “Defaunation resin” (Fig. 3A–E, H, L) and in historical entomological collections will be, in the nearest future, the only suitable records from which to investigate a part of the extinct entomofauna. In this context, the discussion and definition of old and new terms for ancient-Recent resins have the goal of bringing a more accurate framework for the research of living and extinct species preserved as bioinclusions in resin. Furthermore, the definition of Amber Gaps, together with investigation on the causes of resin production^99,100,106^, and on preservation of bioinclusions (e.g.^107^), can help to achieve a better understanding of the relationship between resin production and arthropod entrapment for the study of arthropod diversity through time.

## Materials and methods

### Radiocarbon analyses

The radiocarbon analyses (^14^C) of thirteen samples from six different countries were done by Beta Analytic laboratory: Madagascar (Andranotsara), Tanzania (unknown locality), Colombia (Santander region, unknown locality), Brazil (Roraima) and Dominican Republic (Cotuí), New Zealand (Baylys Beach, Katui, and Waipapakauri). Two samples from New Caledonia (Parc Provincial de la Rivière Bleue) and one from Congo (Mai-Ndombe lake) were analysed by the Radiocarbon laboratory in the University of Barcelona (Dossier no. 2017030808: NL-1536/1538). Results are reported in conventional radiocarbon age (BP), calibrated radiocarbon age (cal. BP) where "present" is defined as 1950 AD, and in calibrated calendar year (cal. AD). Results are reported in “*pMC*” (percent modern carbon) units when the analysed material has more ^14^C than did the modern (1950 AD) reference standard. Samples with plant macroremains were treated with alkali and acid washes in order to remove humic acid and carbonate contamination. Four samples from Madagascar, but without precise location, were dated by Prof. Geyh in 1996; the data are registered in the amber collection at the Senckenberg Research Institute and Museum, Frankfurt (SMF). The material analysed by ^14^C for the present work, and not destroyed by this analysis, is housed at SMF, at University of Barcelona (UB), and at Instituto Geológico y Minero de España (IGME). Other ^14^C data of different samples taken from the literature are presented in the Supplementary Table S1.

### Imaging

Catalogue number and repository of the Defaunation resin and Holocene and Pleistocene copal pieces in Fig. 3 are as follows: Mad-2013-resin-R1-1-213 (A), Mad-2013-resin-R1-156 (B), and IGME without catalogue number (H) are housed at IGME; SMF Be 2563 (D), SMF Be 3724 (F), SMF Be 3786 (G), SMF Be 666 (I), and SMF without catalogue number (J) are housed at SMF; (B), (K), (L), and (E) are housed at UB without catalogue number. Photographs Fig. 3B, C, and H were taken with a digital camera Canon EOS 650D using the software “Macrofotografía” (version 1.1.0.5 https://macrorail.com). Photographs and Z-stacks images of Fig. 3D–G and I–J were produced with a Nikon SMZ25 microscope, using Nikon SHR Plan Apo 0.5× and SHR Plan Apo 2× objectives with a microscope camera Nikon DS-Ri2 and the NIS-Elements software (version 4.51.00 www.microscope.healthcare.nikon.com). Figure 3A was taken with a digital camera Canon EOS 40D. Figure 3E and K–L were taken with a digital camera Canon EOS 70D. Figures 1 and 2 were produced using Adobe Photoshop (CS2, version 9.0 www.adobe.com). Figure 4 was produced using Adobe Photoshop software (CS6, version; 13.0 www.adobe.com).

## Supplementary information

Supplementary information.

## Data Availability

All data needed to evaluate the conclusions in the paper are present in the paper and/or the Supplementary Materials. Correspondence and material related to this paper may be requested from Mónica M. Solórzano Kraemer (monica.solorzano-kraemer@senckenberg.de), Enrique Peñalver (e.penalver@igme.es), and Xavier Delclòs (xdelclos@ub.edu).
