# Supplementary material for: A revised definition for copal and its significance for palaeontological and Anthropocene biodiversity-loss studies
Source: Sci Rep. 2020 Nov 16;10:19904. doi: 10.1038/s41598-020-76808-6 (PMC7669904; doi:10.1038/s41598-020-76808-6)
Supplement: Supplementary file 1 — Supplementary information. [file 41598_2020_76808_MOESM1_ESM.docx]

**Supplementary Information for**

**A revised definition for copal and its significance for palaeontological and Anthropocene biodiversity-loss studies**

**Mónica M. Solórzano-Kraemer^*^, Xavier Delclòs, Michael S. Engel, Enrique Peñalver**

*e-mail: monica.solorzano-kraemer@senckenberg.de

**Supplementary Table: S1**

**Supplementary references for Text Figure 2**

**References**

**Supplementary Table: S1**

**Table S1.** ^14^C data of Defaunation resins and Quaternary copals from diverse countries. Abbreviations: conventional radiocarbon age before present (BP) where "present" is defined as 1950 AD, calibrated calendar year (cal. AD), calibrated radiocarbon age (cal. BP), percent modern carbon (*pMC*; in italics), before Christ (BC), Senckenberg Research Institute, Frankfurt (SMF), University of Barcelona (UBAR), Instituto Geológico y Minero de España (IGME). Ages are presented in the Supplementary Table 1 as obtained from the radiocarbon analysis in BP/*pMC* but also in Cal. BC/AD/BP because for some pieces the variability between both given data can be significant.

| **Samples** | **BP/*pMC*** | **Cal. BC/AD/BP** | **Comments** |
| --- | --- | --- | --- |
| **DEFAUNATION RESIN** | | | |
| “Colombian copal”  NHM II 3059 | < 60 | No information | Piece from Santander Province (Colombia); with *Trigonisca schulthessi* (Hymenoptera: Apidae)*,* in Penney *et al.*^1^ (see^2^ for synonymy) |
| “Zanzibar copal” SMF Be 3724 | 80 ± 30 | 1710 to 1720 cal. AD / 240 to 230 cal. BP | Piece arrived at the SMF between 1874 and 1901 labelled as “Zanzibar copal”, but originated from Tanzania |
| “Brazilian copal”  SMF Be 13856 | 90 ± 30 | 1710 to 1720 cal. AD / 240 to 230 cal. BP | Collected by local people in the Roraima region in agricultural soil without precise location |
| “Tanzania copal” | 115 ± 0.8 | No information | Collected at Moa, Tanzania. Submitted by Dieter Schlee. Clearly discernible nuclear weapons influence indicates very young age of resin, in Felber^3^ |
| Four “Malagasy copal” samples | *115* to *130 pMC* | 1958 to 1989 and 1961 to 1979 cal. AD | Acquired by Mr. Wunderlich and dated by Prof. Geyh in 1996 in the Niedersächsisches Landesamt für Bodenforschung, in Wunderlich^4^ |
| “Malagasy resin”-pit Q1 | *140.47 ± 0.52 pMC* | 1973 to 1975 cal. AD | Collected by MSK, EP and XD in 2017 in Andranotsara (Sambava), in a pit named Q1. It was collected at 15–30 cm depth. Location S14^o^ 38’ 47.2’’ - E50^o^ 12’ 46.7’’, in Delclòs *et al*.^5^ |
| “Cotuí copal”  SMF Be 13858 | 160 ± 30 | 1797 cal. AD to post. 1950 / 153 cal. BP to post. cal. BP | Collected by MSK, EP and XD in 2019 in Zambrana Arriba, close to Cotuí (Dominican Republic), in agricultural soil. Location N18º 56’ 14’’ - W70º 08’ 16’’ |
| “Malagasy resin”-  pit Q2 | *180.83 ± 0.41 pMC* | 2000 to 2004 cal. AD | Collected by MSK, EP and XD in 2017 in Antampolo (Sambava), in a pit named Q2. It was collected at 30–70 cm depth. Location S14^o^ 43’ 4.68’’ - E50^o^ 12’ 8.60’’, in Delclòs *et al.*^5^ |
| “New Caledonia copal” (UBAR, without catalogue number) | 130 ± 25 | 1805 cal. AD to post. cal. BP | Collected by MSK, EP and XD in 2016 in the Parc Provincial de la Rivière Bleue, New Caledonia. Location S22º 07’ 54’’ - E166º 38’ 51’’. The resin was found at 50 cm depth. Without inclusions |
| New Caledonia wood (UBAR, without catalogue number) | 200 ± 40 | 1653 to 1817 cal. AD | Collected by MSK, EP and XD in 2016 in the Parc Provincial de la Rivière Bleue, New Caledonia, resin was collected at 50 cm depth. Location S 22º 07’ 54’’ - E 166º 38’ 51’’. The age of this sample indicates the age of the associated resin |
| “Cotuí copal”  VRI-819 | < 200 | No information | Copal near surface in soil, near Cotuí. Location (N19° 04’- W70° 11’, San Brano, in Felber^7^) probably Zambrana. Collected in 1982 by local inhabitants, submitted by Dieter Schlee from the Staatliches Museum für Naturkunde Stuttgart (SMNS), in Felber^7^ |
| “New Zealand copal” (IGME, without catalogue number) | 200 ± 30 | No information | Piece bought by EP and XD in 2010 in “Katui Kauri Gum Store” as “copal” from the swamp of Katui area, having abundant Recent bioinclusions |
| “Colombian copal”  VRI-820 | < 220 | No information | Copal from unknown locality. Collected in 1982 by local inhabitants, submitted by Dieter Schlee; in Felber^7^ |
| **HOLOCENE COPAL** | | | |
| “Colombian copal”  (without catalogue number) | 140 ± 30 | 1675 to 1735 cal. AD/ 275 to 215 cal. BP | Acquired by a private collector and sold to V. Cattersel; the piece contains a bee identified as *Trigonisca* sp. (Hymenoptera: Apidae). Housed at the University of Antwerp |
| “Costa Rican copal” | 130 ± 30 | 1670 to 1780 cal. AD | Piece from San Cristóbal de Savegre, Puntarenas Province (Costa Rica); in Alvarado *et al.*^8^ |
| “Brazilian copal”  SMF Be 13857 | 140 ± 30 | 1695 to 1725 cal. AD/ 255 to 225 cal. BP | Collected by local people in the Roraima region in agricultural soil without precise location |
| “East African copal” BM 2211(ref.  BM-60864) | 140 ± 50 | 1750 AD | Collected by Sir Joseph Banks from Natural History Museum in London. Piece from Tanzania. Location ca S7° - E38°., in Ambers *et al*.^6^ |
| “Malagasy copal”  SMF Be 13858 | 300 ± 30 | 1645 to 1685 cal. AD/ 305 to 265 cal. BP | Collected in Andranotsara, in a hole made by local people in 2016; it was collected at 50 cm depth. Location S14^o^ 38’ 4.72’’ - E50^o^ 12’ 4.67’’ (close to pit Q1 in Delclòs *et al.*^5^ |
| “Sulawesi copal“  S4-57 | 790 to 700 | No information | Piece of copal from Sulawesi (Indonesia) with beetles of the genus *Tenomerga* (Coleoptera: Archostemata); in Büsse *et al.*^9^ |
| “Zanzibar copal”  SMF Be 3501 | 1,050 ± 30 | 987 to 1051 cal. AD/ 963 to 899 cal. BP | The piece arrived at the SMF between 1874 and 1901 labelled as “Zanzibar copal” but originated from Tanzania |
| “Cotuí copal” | 1,170 ±130 | No information | Piece from Cotuí (Bayaguana region, Dominican Republic); in Wunderlich [4] |
| “Colombian copal” | 1,736 ± 35 | No information | Piece analyzed by D. Grimaldi; in Penney *et al.*^10^ |
| “Colombian copal”  (without catalogue number) | 2,180 ± 30 | 234 to 66 cal. BC/ 2,183 to 2,015 cal. BP | Acquired by a private collector and sold to the family Oermann. The piece contains a nest of termites and is in permanent loan at SMF |
| “Angola copal”  UCLA-686 | 2,830 ± 80 | 880 BC | Copal collected at 90 cm depth in Kalahari sand, Camissombo Plain, NE Angola. Location S8° 9’ - E21° 41’. Collected by J. V. Martins, in Berger *et al*.^11^ |
| “Sulawesi copal“  S4-58 | 4,030 to 3,900 |  | Piece of copal from Sulawesi (Indonesia) with *Tenomerga* sp. (Coleoptera: Archostemata), in Büsse *et al.*^9^ |
| “Congo Copal”  UBAR-1432 | 5,480 ± 35 | 4,356 to 4,232 cal. BC | Collected by Joaquim Nogués (UB) in the margin of Mai-Ndombe lake, Congo. Location S2º28’46.38’’- E18º17’35.40’’. Without inclusions |
| “Colombian copal” | 10,612 ± 62 | No information | Piece from Santander Province (Colombia); with *Trigonisca* sp. (Hymenoptera: Apidae), in Penney *et al.*^1^ |
| “Cotuí copal” | 10,820 +3,530/-2,445 | No information | Piece from Cotuí (Bayaguana region, Dominican Republic), in Wunderlich^4^ |
| **PLEISTOCENE COPAL** | | | |
| “New Zealand copal” | 31,120 | No information | From the ground at Awanui, associated with a buried kauri log in the vicinity. Without inclusions, in Lambert *et al.*^12^ |
| “New Zealand copal” | 30,000 | No information | From the surface of the ground at Lake Ohia, associated with kauri logs. Without inclusions, in Lambert *et al.*^12^ |
| “Dominican copal”  VRI-916 | 31,500  +1,700/- 1,400 | No information | Copal of unknown site, Dominican Republic, submitted by Dieter Schlee, from the Staatliches Museum für Naturkunde Stuttgart (SMNS). No mention about inclusions, in Felber^7^ |
| “Mizunami copal”  VRI-830 | 33,100 +2,000/-1,600 | No information | Collected ca 45 km East of Gifu. Location N35° 37’ - E136° 46’, Honshu I (Japan), in Felber^7^ and Grimaldi^13^. Some inclusions in Aoki^14^, Hiura and Miyatake^15^, and Yosii^16^ |
| “Dominican copal”  D 001; BM-2235 | >36,000 | No information | Piece from Helen Fragnet from the Dominican Republic. Location ca N19° - W69°. With inclusions, in Ambers *et al.*^6^ |
| “New Zealand copal” | 37,000 | No information | From Trig Road in Houhora north of Kaitaia on Ninety Mile Beach from a peat deposit in the sand dunes. Without inclusions, in Lambert *et al.*^12^ |
| New Zealand wood associated with copal (without catalogue number) | 39,640 ± 970 | No information | Collected by EP and XD in 2010 in Waipapakauri. Location S35º 00’56’’ - E173º 11’ 9’’. The age of this sample indicates the age of the copal, which were abundant in the dated wood |
| “New Zealand copal”  (without catalogue number) | 41,370 ± 2,590 | No information | Collected by EP and XD in 2010 in Ripiro beach at Baylys Beach. Location S35º 54’ 52.9’’ - E173º 42’ 17.4’’, near Dargaville (Northland) (Fig. 3). Without inclusions |

**Supplementary references for Text Figure 2**

Ages in the Text Figure 2 **“ Representation of the Amber Bioinclusions Gaps (ABGs) during the Cenozoic, including the Latest Amber Bioinclusions Gap, the newly defined Defaunation resin, and recalibration for the terms copal and amber”** are based on: Pleistocene Mizunami copal^7^, Holocene Colombian copal and Defaunation Colombian resin (^17^, and present work), Holocene East African copal and Defaunation East African resin (including Tanzania and Zanzibar) (^3,6^, and present work), Holocene Dominican copal and Defaunation Dominican resin (^6,49 ,^ and present work), Holocene “Malagasy copal” or Malagasy copal and Defaunation Malagasy resin^5^, Pleistocene New Zealand copal and Defaunation New Zealand resin (^12^, and present work), Bornean amber^18^, Amazonian amber (Peru)^19^, Zhangpu amber (China)^20^, Mexican amber^21,22^, Dominican amber^23^, Venezuelan amber^24^, Ethiopian amber^25,26^, New Zealand amber^27^, Baltic amber^28^, Bitterfeld amber (Germany)^29^, Rovno amber (Ukraine)^30^, Anglesea amber (Australia)^31^, Hat Creek amber (Canada)^32^, Oise amber (France)^33^, Fushun amber (China)^34^, Claiborne amber (Arkansas, USA)^35^, Cambay amber (India)^36^, and Tasmanian amber (Australia)^31^. See Mudelsee *et al.*^37^ for more information about the events indicated with arrows along the upper axis; mPWP from 3.264 to 3.025 Ma (see Haywood *et al.*^38^).

**References**

1. Penney, D. *et al*. Absence of ancient DNA in sub-fossil insect inclusions preserved in ‘Anthropocene’ Colombian copal. *PLoS ONE* **8**(9), e73150; [DOI.org/10.1371/journal.pone.0073150](https://doi.org/10.1371/journal.pone.0073150) (2013).
2. Engel, M. S. *et al*. Nest architecture, immature stages, and ethnoentomology of a new species of *Trigonisca* from northern Colombia (Hymenoptera: Apidae). *Am. Mus. Novit.* **3942**, 1–33 (2019).

Felber, H. Vienna Radium Institute Radiocarbon dates XVI. *Radiocarbon* **29**(3), 389–396 (1987).

1. Wunderlich, J. *Spinnenfauna gestern und heute* (Erich Bauer Verlag bei Quelle and Meyer, Wiebelsheim, 1986).
2. Delclòs, X., Peñalver, E., Ranaivosoa, V. & Solórzano-Kraemer, M. M. Unravelling the mystery of the “Madagascar copal’: age, origin and preservation of a recent resin. *PLoS ONE* **15**(5), e0232623; [DOI.org/10.1371/journal.pone.0232623](https://doi.org/10.1371/journal.pone.0232623) (2020).
3. Ambers, J., Matthews, K. & Burleigh, R. British Museum natural radiocarbon measurements XVIII. *Radiocarbon* **27**(3), 508–524 (1985).

Felber, H. Vienna Radium Institute Radiocarbon dates XIV. *Radiocarbon* **26**(3), 441–448 (1984).

1. Alvarado, G. E., Brenes, J. & Brenes, J. La presencia de ámbar y sub-ámbar (copal) en Costa Rica: El caso de San Cristóbal de Savegre. *Rev. Geol. Am. Central* **49**, 63–72 (2013).
2. Büsse, S. *et al*. Note on using nuclear 28S rDNA for sequencing ancient and strongly degraded insect DNA. *Entomol. Sci.* **20**(1), 137–141 (2017).
3. Penney, D., Green, D. I., Titchener, B. G., Brown, T. A. & Preziosi, R. F. An unusual palaeobiocoenosis of subfossil spiders in Colombian copal. *Arachnology* **15**(7), 241–244 (2012).
4. Berger, R., Fergusson, G. J. & Libby, W. F. UCLA Radiocarbon Dates IV∗. *Radiocarbon* **7**, 336–371 (1965).
5. Lambert, J. B., Johnson, S. C., Poinar, Jr. G. O. & Frye, J. S. Recent and fossil resins from New Zealand and Australia. *Geoarchaeology* **8**(2), 141–155 (1993).
6. Grimaldi, D. A. *Amber:* *Window to the past* (Harry N. Abrams, Inc., in association with the American Museum of Natural History, New York, 1996).

Aoki, J. On the fossil mites in Mizunami amber from Gifu Prefecture, Central Japan. *Bull. Mizunami Fossil Mus.* **1**, 397–399 (1974) [in Japanese with English summary].

Hiura, I. & Miyatake, Y. Mizunami amber and fossil insects. 1. On the fossil Arthropoda in Mizunami amber from Gifu Prefecture (Pleistocene). *Mizunami Fossil Mus.* **1**, 385–392 (1974).

Yosii, R. Fossil Collembola contained in the Mizunami amber (Insecta: Collembola). *Mizunami Fossil Mus.* **1**, 409–411 (1974).

1. Penney, D. Sub/fossil resin research in the 21st Century: trends and perspectives. *PalZ.* **90**, 425–447 (2016).
2. Kocsis, L. *et al*. The Bruneian record of “Borneo Amber”: A regional review of fossil tree resins in the Indo-Australian Archipelago. *Earth-Sci. Rev.* **103005**; DOI.org/10.1016/j.earscirev.2019.103005 (2019).
3. Antoine, P. O. *et al*. Amber from western Amazonia reveals Neotropical diversity during the middle Miocene. *Proc. Natl. Acad. Sci. USA* **103**(37), 13595–13600 (2006).
4. Shi, G., Dutta, S., Paul, S., Wang, B. & Jacques, F. M. B. Terpenoid Compositions and Botanical Origins of Late Cretaceous and Miocene Amber from China. *PLoS ONE* **9**(10), e111303; [DOI.org/10.1371/journal.pone.0111303](https://doi.org/10.1371/journal.pone.0111303) (2014).
5. Solórzano-Kraemer, M. M. Systematic, palaeoecology, and palaeobiogeography of the insect fauna from Mexican amber. *Palaeontographica Abt. A* **282**, 1–133 (2007).
6. Serrano-Sánchez, M. L. *et al*. The aquatic and semiaquatic biota in Miocene amber from the Campo La Granja mine (Chiapas, Mexico): Paleoenvironmental implications. *J. S. Am. Earth Sci.* **62**, 243–256 (2015).
7. Iturralde-Vinent, M. A. & Macphee, R. D. Remarks on the age of Dominican amber. *Palaeoentomology* **2**(3), 236–240 (2019).
8. Pérez, L. M. *et al*. Palaeontology, sedimentology, and biostratigraphy of a fossiliferous outcrop of the Early Miocene Querales Formation, Falcón Basin, Venezuela. *Swiss J. Palaeontol.* **135**(2), 187–203 (2016).
9. Perrichot, V. *et al*. The age and paleobiota of Ethiopian amber revisited. *IPC5 - 5th International Palaeontological Congress* Abstract 23 (2018).
10. Bouju, V. & Perrichot, V. A review of amber and copal occurrences in Africa and their paleontological significance. *BSGF - Earth Sci. Bull.* **191**(17); [doi.org/10.1051/bsgf/2020018](https://doi.org/10.1051/bsgf/2020018) (2020).
11. Schmidt, A. R. *et al*. Amber inclusions from New Zealand. *Gondwana Res.* **56**, 135–146 (2018).
12. Kasiński, J. R., Kramarska, R., Słodkowska, B., Sivkov, V. & Piwockl, M. Paleocene and Eocene deposits on the eastern margin of the Gulf of Gdańsk (Yantarny P-1 bore hole, Kaliningrad region, Russia). *Geol. Q.* **64**, 20–53 (2020).
13. Dunlop, J., Kotthoff, U., Hammel, J. U., Ahrens, J. & Harms, D. Arachnids in Bitterfeld amber: A unique fauna of fossils from the heart of Europe or simply old friends? *Evol. Syst.* **2**, 31–44 (2018).
14. Perkovsky, E. *et al*. A comparative analysis of the Baltic and Rovno amber arthropod faunas: representative samples. *Afr. Invertebr.* **48**(1), 229–245 (2007).
15. Stilwell, J. *et al*. Amber from the Triassic to Paleogene of Australia and New Zealand as exceptional preservation of poorly known terrestrial ecosystems. *Sci. Rep.* **10**, 5703 (2020).
16. Poinar, Jr. G. O., Archibald, B. & Brown, A. New amber deposit provides evidence of early Paleogene extinctions, paleoclimates, and past distributions. *Can. Entomol.* **131**, 171–177 (1999).
17. Nel, A. & Brasero, N. Oise amber in *Biodiversity of fossils in amber from the major world deposits*, D. Penney, Ed., pp. 137–148 (Siri Sci. Press, Manchester, 2010).
18. Wang, B. A diverse paleobiota in Early Eocene Fushun amber from China. *Curr. Biol.* **24**(14), 1606–1610 (2014).
19. Saunders, W. B., Mapes, R. H., Carpenter, F. M. & Elsik, W. C. Fossiliferous amber from the Eocene (Claiborne) of the Gulf coastal plain. *Geol. Soc. Am. Bull.* **85**(6), 979–984 (1974).
20. Rust, J. *et al*. Biogeographic and evolutionary implications of a diverse paleobiota in amber from the early Eocene of India. *Proc. Natl. Acad. Sci. USA* **107**(43), 18360–18365 (2010).
21. Mudelsee, M., Bickert, T., Lear, C. H. & Lohmann, G. Cenozoic climate changes: A review based on time series analysis of marine benthic δ18O records. *Rev. Geophys.* **52**(3), 333-374 (2014).
22. Haywood, A. M., Dowsett, H. J. & Dolan, A. M. Integrating geological archives and climate models for the mid-Pliocene warm period. *Nat. commun.* **7** (1), 1–14 (2016).
